# Learning-Based Motion-Intention Prediction for End-Point Control of Upper-Limb-Assistive Robots

**DOI:** 10.3390/s23062998

**Published:** 2023-03-10

**Authors:** Sibo Yang, Neha P. Garg, Ruobin Gao, Meng Yuan, Bernardo Noronha, Wei Tech Ang, Dino Accoto

**Affiliations:** 1 School of Mechanical and Aerospace Engineering, Nanyang Technological University, Singapore 639798, Singapore; 2 Rehabilitation Research Institute of Singapore (RRIS), Nanyang Technological University, Singapore 308232, Singapore; 3 School of Computer Science and Engineering, Nanyang Technological University, Singapore 639798, Singapore; 4 Department of Mechanical Engineering, Robotics, Automation and Mechatronics Division, KU Leuven, 3590 Diepenbeek, Belgium

**Keywords:** upper limb assistive robots, wearable sensors, motion intention detection, human–robot interaction, sensory fusion, machine learning

## Abstract

The lack of intuitive and active human–robot interaction makes it difficult to use upper-limb-assistive devices. In this paper, we propose a novel learning-based controller that intuitively uses onset motion to predict the desired end-point position for an assistive robot. A multi-modal sensing system comprising inertial measurement units (IMUs), electromyographic (EMG) sensors, and mechanomyography (MMG) sensors was implemented. This system was used to acquire kinematic and physiological signals during reaching and placing tasks performed by five healthy subjects. The onset motion data of each motion trial were extracted to input into traditional regression models and deep learning models for training and testing. The models can predict the position of the hand in planar space, which is the reference position for low-level position controllers. The results show that using IMU sensor with the proposed prediction model is sufficient for motion intention detection, which can provide almost the same prediction performance compared with adding EMG or MMG. Additionally, recurrent neural network (RNN)-based models can predict target positions over a short onset time window for reaching motions and are suitable for predicting targets over a longer horizon for placing tasks. This study’s detailed analysis can improve the usability of the assistive/rehabilitation robots.

## 1. Introduction

Motion impairment of the upper limbs, occurring, for example, due to a stroke, or total lack of motion due to amputation, can seriously affect the performance of activities of daily living (ADL) [1,2]. Assistive robots such as exoskeletons [3] and intelligent prostheses [4] are designed to assist patients in daily activities and help to improve their quality of life [5]. However, the current active assistive robots are still not widely accepted by users due to the lack of a reliable and intuitive controller which can detect the user’s intention and provide assistive motion in a natural way [6].

**Figure 1:**
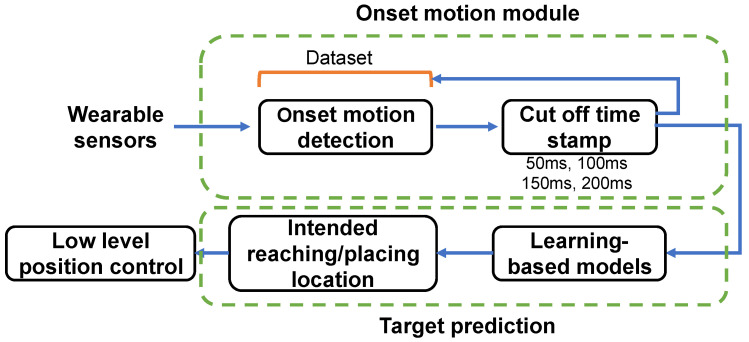
Overview of the proposed learning-based controller. The onset motion data are acquired from different sensory interfaces. The input features are then computed for four onset time windows of motion. Then, the processed motion samples are given as input to multiple learning-based intention prediction models for end-point prediction. Finally, the target end-point positions will be given as a reference point to the low-level controller for the robot to move.

Unlike the cyclic motion of the lower limb, the motion of the upper limb has more joint redundancies and interactive functionalities with environments. Therefore, it is challenging to intuitively predict the motion intention of the upper limb while simultaneously driving its multi-DOF joints. In the state of the art, two main approaches of motion intention detection are explored: human-motion activity recognition [7] and continuously decoding joint angles in time series [8]. However, the methods covered above could not accurately and intuitively recognize the intended reaching goal of humans’ upper limbs in planar space and thus cannot be used to provide assistance in reaching a specific goal. Therefore, we propose an intuitive control system that can directly predict the end-effector position in a planar space from the user’s onset motions (Figure 1), which enables one to encode of a large amount of information about the trajectory [9]. Then, an inverse kinematics model can be used to derive the relative reference joint angles. The detection of the final goal based on onset motion can be useful in achieving the relevant motion assist function without too much of a mental burden on the patients [10].

State-of-the-art works [10] which can predict the user’s goal in a planar space from their onset motion use various sensors, such as eye gaze and EMG to learn a prediction model from user data. However, it is not clear which sensors are necessary to make such a prediction. Therefore, in this work, we explored combinations of various sensors, such as EMG, IMU, MMG, and various learning algorithms; and evaluated which sensors and algorithm combinations provide the best accuracy. Interestingly, our results show that only the IMUs are sufficient to predict the final position when using learning algorithms such as LSTM/GRU/RNN, which have an inductive bias for sequential data. This finding could be useful in improving the usability of assistive robots by simplifying the sensing system.

The main contributions of this paper are the following: (1) A control system is proposed to utilize the onset motion to predict the user’s target reaching or placing location. The system combines several sensory interfaces and adds a novel motion intention detection module. (2) A motion dataset was constructed which can predict the intended motion of cross-subjects. Multiple machine learning models were evaluated under certain onset time windows of motion. RNN-based models provided relatively high prediction performance of the end-point position. (3) We evaluated the performances of different combinations of sensors on the reaching and placing motions and discovered that adding EMG and MMG sensors over IMU sensors does not increase prediction accuracy.

## 2. Background

### 2.1. Control Based on Physiological Signals

Physiological signals are widely used as control interfaces for assistive and rehabilitation robots, which can detect the potential intention of motion [11,12]. They mainly measure the electrical activity of muscles, known as EMG signals [13], or the mechanical response of muscles, known as MMG signals [14]. Myoelectric control interfaces using EMG signals can provide information about the intention of motion 50–100 ms before the actual motion happens [15]. One popular method is to recognize the motion pattern, in which EMG signals from different areas of muscles can detect the motion pattern, such as shoulder elevation and abduction [16]. However, the main limitation of this kind of approach is that only recognizing the movement patterns is not sufficient for assisting in ADLs accurately. There is no interaction between humans and the environment which cannot receive particular assistance. In addition, researchers have worked on predicting kinematic joint angles of the upper limb in time series using learning-based methods [17] or model-based methods (e.g., musculoskeletal model) [18]. Ref. [17] proposed a neural network to continuously predict multi-joint angles. Each joint angle’s root means square error (RMSE) was on average 9.16${}^{\circ }$ per selected time window, which could lead to significant errors between the subject’s intended location and the predicted location.

On the other hand, MMG-based control interfaces are robust to changes in skin impedance and require simple calibration and placement procedures. Its effectiveness has been demonstrated in stroke rehabilitation [19], human–robot interaction [20], and prosthetic control [21,22]. In order to compensate for the limitations of EMG, some approaches combined these signals as a hybrid physiological modality. For example, hybrid EMG-MMG sensing was proposed to classify the motion pattern of the hand [12]. The authors showed that the classification accuracy was enhanced by 8.8% compared to only using EMG signals.

### 2.2. IMU Control Interfaces

IMU signals are used to predict the joint information of the upper limb, such as joint angles and velocities. Normally, a mapping model can be established to match the relationship between past movement and intended motion [23]. A common method is to use human kinematic movement to predict the motion pattern. For example, researchers investigate the inertial sensing information to classify the reaching or grasping motion for stroke patients [24]. On the other hand, Huang et al. [25] used two IMUs for predicting intended joint motion in real-time via deep-recurrent neural networks. The results show that the model can achieve a prediction error of ±2.93 degrees within a predicted horizon of 50 ms. However, this work only predicted the motion of the knee joint, which has fewer motion redundancies than the upper limb.

### 2.3. Multi-Modal Sensing Interfaces

In these techniques, multiple signals are fused so that the shortcomings associated with each signal are compensated by complementing the intention prediction [26,27]. The concept of multi-modal sensing interfaces has shown improved accuracy, reliability, and robustness compared to single-signal methods [28]. Fusing EMG and EEG data provided a reliable source of data for creating a brain–computer interface able to decode the motion pattern of elbow flexion (one DOF), even under conditions of muscular fatigue [29].

To date, a commonly used approach is to fuse physiological sensors (e.g., EMG sensors) and kinematic sensors (e.g., IMU). EMG signals cannot provide direct kinematic information about limb motion, which can be provided by kinematic sensors such as IMUs. Little et al. [30] used a multi-modal wearable system, including EMG, IMU, and a stretch sensor, to predict elbow joint angular trajectory. The different time windows of onset motion were input to ten machine learning algorithms, showing that combining multiple features have better performances than single features. Williams et al. [31] proposed a recurrent convolutional neural network for position-aware myoelectric prosthesis control strategies, which fused EMG and IMU signals. However, the common limitation of the mentioned studies is that prediction is limited to a single joint, which does not correspond to the full movement of the upper limb in ADLs. Ren et al. [8] fused the features from EMG and IMU as input to predict the upper limb of multi-joints. The fused signals can reduce the predicted errors and the delay time of movement between the human and the robot compared with using only IMUs. However, the main limitation of this work is that it only synchronizes human motion and robot. It still requires humans to provide sufficient control effort during the motion trajectories.

## 3. Methods

### 3.1. Participants

Five healthy participants (four males, one female, 27.8±1.2 years old) with no previous history of musculoskeletal problems participated in this experiment. All were right-handed and used their dominant hands in the experiment. This experiment was approved by the Institutional Review Board of Nanyang Technological University (IRB-2020-10-006). All the subjects read and signed a consent form before the experiment. The subjects were not physically constrained but instructed to perform the motions according to the experimental protocol. There is not much variance in the sensor signals from different humans [32]. Hence, we recruited five individuals for the data analysis and the testing of algorithms.

### 3.2. Experimental Setup and Protocol

**Figure 2:**
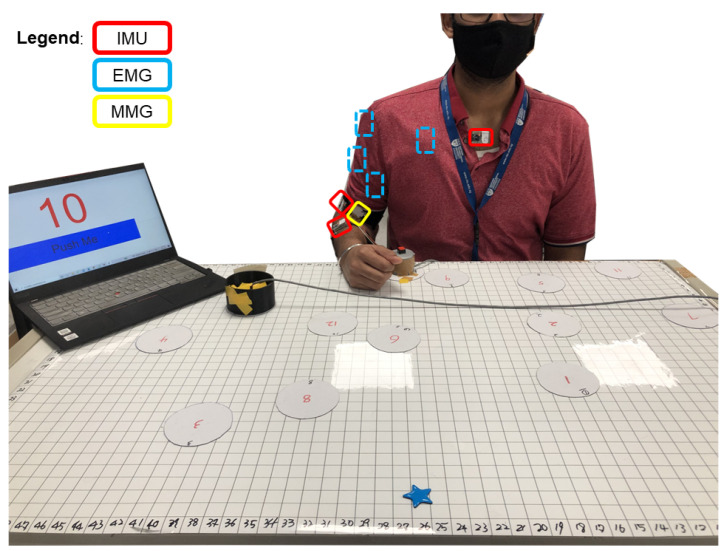
**Experimental setup:** The subject sat in front of the experimental table. Then, he moved the cup according to the number of the positions indicated on the screen of a laptop.

**Figure 3:**
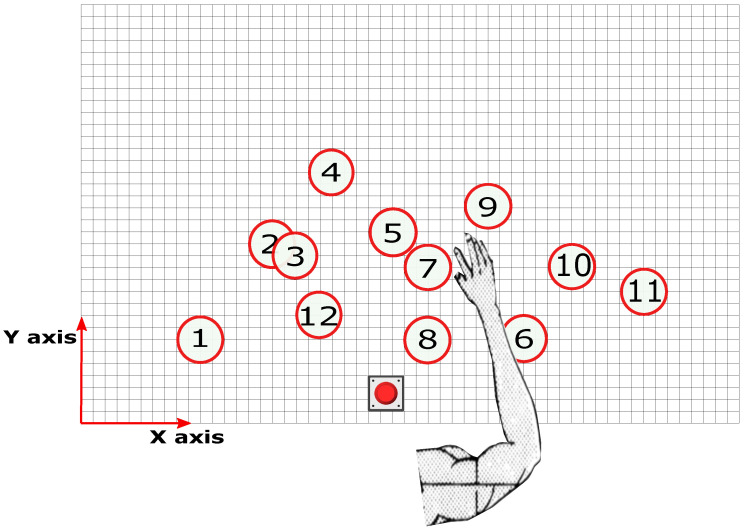
**Schematic of the experimental table:** Each red position is made up of a 4 cm radius circular. Twelve magnetic stickers are placed in different locations.

The participants were asked to sit in front of a table covered by a 110×70 cm rectangular magnetic soft pasteboard, divided into a 55×35 grid composed of 2×2 cm squares (Figure 2). The participants were then instructed to move a cup between 12 different positions located on the board (Figure 3). The positions were randomly generated and marked on the board by circular magnetic stickers. Each of these numbers will be repeated in groups of ten. To aid in this, a Cartesian coordinate system with its origin at the bottom left corner of the board was considered. All positions were within the maximum reachable radius of each subject. Each position was assigned a number, and the subject was informed of the sequence of positions by following instructions on a PC. Each experimental session included 120 position numbers. The same position cannot be moved twice in a row. An analog button, named the "home" button, was placed in front of the participant and was used to signal the beginning and end of each cyclic motion. Another button was placed under the cup, such that it was pressed when the cup was on the table, for later aiding in data segmentation.

The protocol for each motion was as follows: (1) push the “home” button, signaling the beginning of the motion; (2) place the cup on the instructed location; (3) move the hand back to the starting point and push the “home” button, signaling the end of the motion. The main reason for selecting the reaching and placing motion types as the motions for experimental analysis was that these two motions are the two most basic and most used movements in daily life. Each cycle was repeated 240 times, divided over two sessions (120 each). Each session took approximately 15 min, and there was a 5 min rest in between. There were no physical constraints on the upper limb throughout the performance of the experiments.

### 3.3. Data Collection

**Figure 4:**
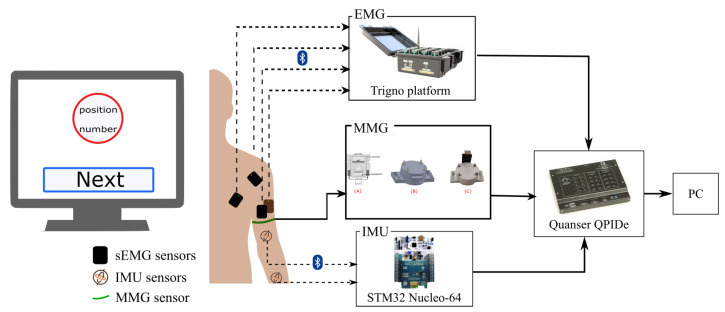
**Data acquisition flow:** The IMUs and the EMG and MMG sensors were placed on the upper limb of the participant. The EMG and IMU data were transmitted wirelessly, and the MMG data were captured via a wired connection. A DAQ acquires the data (Quanser PIDe) connected to a PC.

The data collection setup was established as shown in Figure 4. Four EMG sensors (Delsys Trigno) were placed on the muscle belly of the biceps brachii (BB), triceps brachii (TB), anterior deltoid (AD), and pectoralis major (PM) following the SENIAM guidelines [33].

Two wireless IMU sensors (ST Microelectronics STEVAL-STLKT01V1) were attached with elastic hook-and-loop tape to the dorsal side of the forearm and the dorsal side of the upper arm. The sensors communicate via low-energy Bluetooth (BLE) with a development board (STM32 Nucleo-64) equipped with an expansion board (X-NUCLEO-IDB05A1) to allow Bluetooth communication. In addition, the single self-designed mechanomyography (MMG) sensor was attached to the BB using the Velcro strap. The MMG sensor used in this experiment included two components: one was a microphone, and the other part was a conical acoustic chamber that can be attached to the microphone. It enables one to measure the low-frequency mechanical vibrations generated by the contractions or oscillations of muscles [34]. All three types of wearable sensors connected to a data acquisition board (Quanser PIDe) to trigger the synchronization at 100 Hz.

### 3.4. Data Processing

#### 3.4.1. Segmentation

All data from the three types of sensors were segmented into three motion patterns: reaching, placing, and homing. Specifically, reaching refers to the subject’s hand moving from the home position to the instructed target location; placing corresponds to the participant lifting up the cup and moving it to the next instructed location; moving back corresponds to moving the hand back to the initial position. Each motion pattern contains the motion trajectories of participants, which cover 1200 independent, unique trajectories.

Segmentation was performed based on the streamed binary data from the “home” button and the button under the cup. The “home” button separates each repetition of the cyclic motions. The cup button transmits either “0” when the cup is not on the table or “1” when the cup is placed down. A signal of “0” corresponds to placing movements, whereas a signal of “1” corresponds either to reaching or moving back. The distinction between the latter two movements is done through the signal of the “home” button: reaching occurs between a triggering of the home button and the start of a signal of 1 in the cup button; placing occurs between the end of a signal of 1 in the cup button and the triggering of the home button.

#### 3.4.2. Feature Extraction

The input features used for model training were extracted from the processed and segmented EMG, IMU, and MMG data. The objective of this experiment was to use the onset movement to predict the task goal in the planar space. In a sense, it can be classified as an inference of the task goal by a short-term motion [35]. Therefore, the input dataset was extracted on the four onset time windows (OTW), listed as 50, 100, 150, and 200 ms.

**Figure 5:**
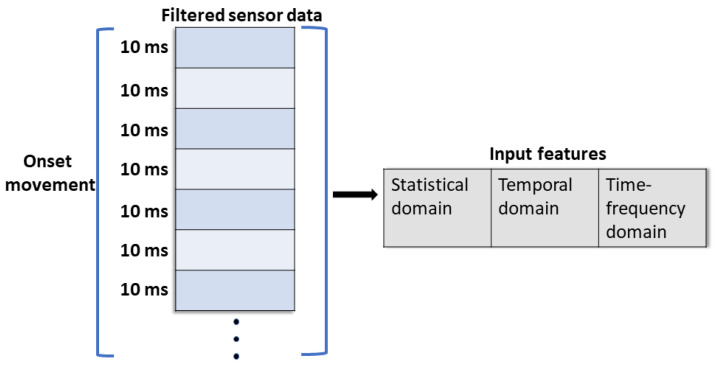
The input features are calculated within the whole time window of onset movement. The motion data of each subject can be reformed as a single vector.

The former normally requires feature engineering to enhance the model’s accuracy. Here, the filtered sensory data were subsequently calculated within the sequential input time window, shown in Figure 5. The onset motion data of each subject can be reformed as a single vector. The extracted features can be divided into the statistical domain, the temporal domain, and the time-frequency domain. There were three selected statistical-domain features consisting of mean absolute value (MAV), root-mean-square (RMS), and variance (VAR), and one temporal feature, waveform length (WL), [36,37,38], are calculated as follows:

(1) $$\begin{array}{cc} MAV & =\frac{1}{n}\sum_{i=1}^{n}\left|x_{i}\right| \end{array}$$

(2) $$\begin{array}{cc} RMS & =\sqrt{\frac{1}{n}\sum_{i=1}^{n}{\left(x_{i}\right)}^{2}} \end{array}$$

(3) $$\begin{array}{cc} VAR & =\sqrt{\frac{1}{n}\sum_{i=1}^{n}{\left(x_{i}-\bar{x}\right)}^{2}} \end{array}$$

(4) $$\begin{array}{cc} WL & =\sum_{i=1}^{n-1}\left|x_{i+1}-x_{i}\right| \end{array}$$

where *x* represents the values of specific signals (IMU, EMG, and MMG), *n* refers to the number of onset motion samples, and *i* is the time steps. Here, i+1 refers to a 10 ms increment. Concerning physiological features, there are five channels for the EMG and MMG sensors. Based on the four selected statistical features, there are 4∗5=20 features extracted from EMG and MMG in the statistical domain In contrast, the signals of IMUs are all kinematic data related to upper-limb motion. Two IMUs can output eight quaternions, where each generates four numbers (*x*, *y*, *z*, and *w*). In addition, the relative angles of the elbow joint and shoulder joint are derived from quaternions, which include five joint angles in total. For all the above-mentioned values, including physiological features and kinematic features, we averaged each of them and used them as features. Overall, the total number of features was 20+8+10=38 features, where 20 refers to all physiological features; 8 refers to features of quaternion generated from two IMUs; and 10 refers to kinematic data generated from positions, velocities, and accelerations of joint angles. Regarding the time-frequency domain, the short-time Fourier transform (STFT) was applied to the physiological signals. The window function was chosen as the “Hanning window”; the window segment length was set to 5, and the overlapping window length was set to 2. Hence, the numbers of sEMG and MMG features were $5*n_{otw}$, where 5 refers to four EMG sensors and one MMG sensor. $n_{otw}$ was the length of each onset motion time window.

RNN methods are normally end-to-end approaches which can input raw data to train and test the model. Therefore, extraction of features is not necessary for this type of model, allowing for the filtered sensor data of each subject to be directly input to the RNN models.

#### 3.4.3. Standardization

The input samples were standardized by removing the mean and scaling to unit variance.

### 3.5. Models

In this study, we designed and compared 5 machine learning regression algorithms and three recurrent neural networks. Training and testing were done on a computer with an NVIDIA Quadro P5000, an Intel Xeon processor, and 32 GB RAM. We used the Sklearn 1.0.1 and Pytorch 1.12 libraries to design the model. As discussed in our previous work [30], multiple algorithms based on four different working principles were selected for performance comparison. All the models were used to predict hand position. The details of the models are further described in the following subsections.

#### 3.5.1. Tree-Based Models

Tree-based regression models enable one to build a tree-like structure for predicting the output [39]. In this study, the decision tree model, extra trees model, and the random forest model (RF) were selected for training and testing the dataset. However, the RF model showed quite low prediction accuracy compared to other algorithms during cross-validation. Hence, RF was discarded during the testing.

#### 3.5.2. Support Vector Regression

A grid search was performed on both *x* and *y* axes to find the best hyperparameters of each SVR. The mean test error was used to evaluate the results, which provide the lowest value corresponding to the best combination of the parameter. The RBF kernel was selected because of the non-linearity of the dataset. The γ was set to 0.001, and the soft margin parameter *C* was 1000.

#### 3.5.3. Feedforward Neural Networks

A multi-layer perceptron (MLP) is a fully connected feed-forward neural network. The grid search was used to determine the best hyperparameters on two MLPs (*x* and *y* axis). The activation function was tanh function, and the hidden layer size was 50. In addition, the learning rate was set to adaptive mode, and the alpha was equal to 0.0001.

#### 3.5.4. Recurrent Neural Networks

Three different RNN models were designed in this study, including RNN, long short-term memory (LSTM) networks, and gated recurrent unit (GRU) networks [40]. All the RNNs consisted of three hidden layers, including two recurrent layers and one fully-connected linear layer. The recurrent layers were set as RNN, LSTM, or GRU, respectively.

The groups of hyper-parameters are listed as the following content for searching for the best combinations. The list of hidden units was $\left\{24,28,64,128\right\}$. The learning rate was set to 0.001. The decay rate of weight was set to 0. Moreover, the adaptive moment estimation (Adam) was used in this study, which can speed up the convergence of the neural networks. The batch size represents the number of samples input simultaneously into the training models before updating weights, which was set as 32. The list of epoch numbers was $\left\{300,350,400\right\}$.

All groups of hyper-parameters were tuned to acquire the best combination of the parameter on the validation set.

### 3.6. Output

The reaching and placing tasks were the two categories of movement patterns that were analyzed in this study. The outputs of the prediction models were two values of the *x* axis and *y* axis in the 2D position table. The unit of coordinates is one grid represented as 2 cm.

### 3.7. Cross-Validation

A typical approach of 80% of the dataset for training and 20% for testing was randomly selected. Within the training dataset, 5-fold cross-validation was used to validate the performance of the models and find the best hyperparameters of each model [41]. In the training dataset, 10% of the data were used for validation. Twenty-percent were used as testing data and not involved in cross-validation. The cross-validation was only applied to the remaining 80% of the data. This procedure enabled us to avoid data leakage and overfitting.

### 3.8. Evaluation Metric

The performance of the prediction model was evaluated by comparing the predicted coordinates with the real location of the object. The prediction accuracy was calculated as

(5) $$Accuracy=\frac{N_{correct}}{N_{test}}\cdot 100\%$$

**Figure 6:**
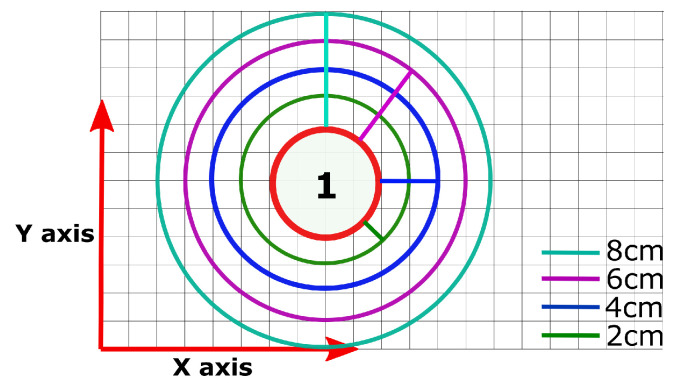
The settled margin of radius error for object’s location. The largest range was chosen to be coincident with the palm-to-wrist length of the 99% of adults, corresponding to 8 cm [42].

In this context, $N_{correct}$ refers to the number of positions accurately predicted in the testing set, and $N_{test}$ refers to the total number of positions in the testing set. Hence, this represents the overall testing result. Four different error ranges were investigated as four circles, illustrated in Figure 6. The radius of each circle is increased by $\Delta R=\left\{2,4,6,8\right\}$, respectively, based on the absolute position (red circle). Since this study only considered joint movements of the elbow and shoulder, the choice of ΔR was dependent on the working range of the wrist. The largest ΔR was chosen to be coincident with the palm-to-wrist length of the 99% of adults, corresponding to 8 cm [42].

## 4. Results

In this section, the selected and proposed learning-based models are compared using the proposed evaluation metric that was introduced in (5). In each subsection, the performances for the four selected onset time windows and working ranges R were analyzed and compared when computed with different models. In addition, we compare the prediction accuracy using different sensors as inputs.

### 4.1. Reaching Task

Table 1 shows the prediction accuracy of the selected ML techniques when input features from all sensors were used, for the reaching task test set. It can be observed that RNN variant models significantly outperform other ML models. Under the condition of ΔR≤ 2 cm, the LSTM prediction model can achieve the highest accuracy rate of 81% with 50 ms OTW. A Wilcoxon signed-rank test was performed and showed that the RNN variant models achieved better prediction performance than the ML models (p=0.00003). However, the RNN model was not significantly different from LSTM and GRU (p=0.203,0.085).

**Figure 7:**
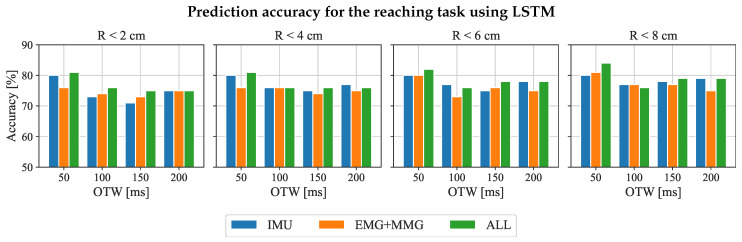
Prediction accuracy of the “reaching” task for different combinations of input features considering all subjects. The performance of the analysis mainly targets the LSTM model. The blue bars represent the results that only use kinematic input features labeled as IMU. The orange bars refer to the physiological features input only, labeled as EMG + MMG. Results with the combinations of input features are shown in green (labeled as all).

Furthermore, we also analyzed the prediction performance based on different input features, which include the features extracted from physiological information (EMG + MMG only), kinematic information (IMU only), and fused information (all features). Based on the previous results, the RNN-based model has higher prediction accuracy than other models with all input features. In addition, the statistical result does not show a significant difference among the three RNN-based models (RNN/GRU/LSTM). Therefore, the LSTM was chosen to analyze the feature importance. Figure 7 shows the prediction accuracy of end-point position based on different input features, which considered the selected four OTWs and radius of constrains. Only using IMU sensors as the input signal can achieve 80% prediction accuracy with 50ms OTW under the condition of ΔR≤ 2 cm, which is only 1% less than the input of all features. In addition, the prediction accuracy decreased with the incrementation of OTW. When only EMG + MMG were considered as the input signal, the performance was worse than the other groups of input features.

### 4.2. Placing Task

Table 2 shows the prediction performance on the selected models when features from all sensors are used, for the placing task test set. The GRU and LSTM models both reach the highest prediction accuracy (83%) for ΔR≤2 cm when the OTW is 200 ms. As the error distances increase, the accuracy of the GRU model can reach 88%. A Wilcoxon test is performed and shows that the RNN variant models are significantly different from the ML models (p=0.00003). However, the RNN model is not significantly different from LSTM and GRU (p=0.857,0.364).

**Figure 8:**
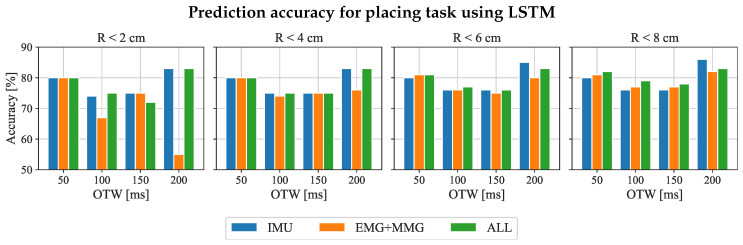
Prediction accuracy of the “placing task” for different combinations of input features for all participants subjects. The LSTM model is the main model to be analyzed in this section. The blue bars represent the results that only use kinematic input features labeled as IMU. The orange bars refer to the physiological features input only, labeled as EMG + MMG. Results with the combinations of input features are shown in green (labeled as all).

We also analyzed the effect of different input features on the prediction results of the LSTM model, which accessed the same procedures as the “Reaching task”. Figure 8 shows that the accuracy using IMU alone is similar when using all sensors. Both of them can reach 83% under 200 ms OTW and the condition of ΔR≤ 2 cm. With the increase in error distance, the prediction accuracy of IMU will continue to improve to 85%, but the performances of all features as input remained at 83%.

## 5. Discussion

The main objective of this study was to validate the performance of the proposed wearable sensing system for detecting human upper limb motion intentions while reaching and placing tasks.

We want to develop a learning-based high-level controller, including the selection of sensory interfaces, prediction algorithms, and onset detection of motion. This high-level controller can be used to control multi-DOF upper-limb-assistive devices, which could help patients to finish daily reaching and placing tasks in planar space. The analysis was done offline but could be performed online in future experiments.

Firstly, multiple prediction algorithms were evaluated on the same testing set. The sequential models outperformed the other selected models in both reaching and placing tasks. The results of the Wilcoxon test showed that the performance of the RNN variant was significantly high among all selected models. Additionally, the training dataset randomly involved motion samples from all subjects, which suggests that this model is subject-independent and enables one to generalize on different subjects. RNN-based models enable one to use their memory states to process temporal sequences of inputs [43]. Therefore, the LSTM model achieves higher prediction performance for reaching and placing motions.

The second hypothesis is that the length of OTW has an impact on the prediction performance of the LSTM model, as shown in Table 1 and Table 2. For reaching tasks, the accuracy of the prediction achieved the highest prediction accuracy (80%) with a 50 ms input feature for RNN-based models. However, the prediction accuracy gradually decreased with the increment in OTW. The potential reason is that the input features mainly refer to the information on the human’s upper limb, which does not include the information on the environmental position. The result suggests that using 50 ms of onset motion data is enough to predict the final reaching position of the upper limb. For placing tasks, OTW with 200 ms onset motion data input to the GRU and LSTM model, which enabled us to achieve 83% prediction accuracy under 2 cm error distance. Therefore, the comparative analysis can suggest that the 200 ms OTW is suitable for the prediction of placing tasks. However, 19% and 17% error rates are still too high for the practical control system. The performance might be improved by proposing a new model for prediction, such as a mixture density network (MDN), which can model confidence in the prediction so that the assistive robot assists only when confidence in prediction is high.

The third hypothesis is that the kinematic features extracted from IMU (mentioned in Section 3.4.2) are sufficient for predicting the desired end-point position of the upper limb. Figure 7 and Figure 8 show that the prediction based on IMU inputs can achieve up to 80% accuracy for reaching tasks and 83% for placing tasks, which can be roughly equal to the prediction accuracy after fusing EMG and MMG input signals. Thus, additional input signals from EMG + MMG do not significantly increase prediction accuracy. In addition, the whole system can become complex after adding EMG and MMG sensors. In practical scenarios, the EMG and MMG signals of stroke patients are different compare with healthy people, so it is tough to generalize the prediction model on stroke patients. Hence, IMU sensors with selected algorithms are possibly a good starting point for stroke patients. In addition, a less complicated wearable sensing system can increase the comfort and convenience of the patient.

## 6. Future Work

There are still some limitations that need to be addressed in future work. Firstly, the prediction accuracy needs to be further improved for online testing. We aim to propose new prediction models, such as the mixture density network (MDN), which might model the uncertainty level of motion based on the IMU input features or the hidden markov model (HMM) [44,45], which enables one to apply sequential modeling to further improve the prediction performance. For instance, the exoskeleton or intelligent prosthesis might execute the assistance when the confidence in prediction is high. Moreover, this strategy is more suitable for doing online testing. Secondly, the different combinations of input features extracted from sensors can result in better performance of prediction accuracy. Hence, we want to approach more types of input features (such as from the time series feature extraction library [36]) to compare the performances of alternatives. In addition, the Time Series Subsequence Search Library (TSSEARCH) also can be implemented to extract features [46]. Thirdly, we intend to implement the whole system in different types of upper-limb-assistive robots (exoskeleton or assistive robotic arm extender) for online testing. The online performance will be evaluated on both healthy subjects and patients. A high-level control system will use our proposed framework to predict the desired end-point position, and the low-level controller will command each actuated joint to position the end-effector to the desired target position using inverse kinematics. Finally, the whole system will be evaluated in an assistive robot with stroke patients.

## 7. Conclusions

In this paper, we proposed a novel human–robot interaction strategy for upper-limb-assistive robots, which can predict the end-point position using human intention in planar space. The proposed multi-modal wearable sensing system was used to extract physiological from EMGs and MMG and kinematic features from IMUs. We analyzed combined methods, including four onset time windows and multiple machine learning models. The results showed that the RNN-based model has better prediction accuracy than other traditional regression models. Moreover, the time-window selection of the proper onset motion can enhance the prediction performance. Most importantly, only using IMUs as a human–robot interface is sufficient to detect human motion intention in this scenario. Therefore, it enables one to simplify the whole control system and be used clinically. The proposed method can improve the usability of assistive robots. While detecting the intended motion of patients, the assistive robots enable helping the user to finish the desired motions smoothly and safely.

## Figures and Tables

**Table 1:** Comparison of prediction accuracy for reaching task using different models, with all sensor data.

Error Distance [cm]	OTW [ms]	Model							
		SVR	DecisionTree	AdaBoost	ExtraTrees	MLP	RNN	GRU	LSTM
ΔR≤2	50	3%	28%	57%	5%	14%	80%	80%	**81%**
	100	4%	21%	62%	6%	17%	71%	75%	**76%**
	150	4%	29%	65%	7%	18%	**75%**	**75%**	**75%**
	200	11%	30%	71%	6%	24%	72%	73%	**75%**
ΔR≤4	50	7%	31%	61%	16%	38%	80%	**81%**	**81%**
	100	14%	26%	66%	15%	46%	**76%**	75%	**76%**
	150	21%	34%	71%	20%	44%	75%	75%	**76%**
	200	26%	33%	74%	26%	52%	**77%**	76%	76%
ΔR≤6	50	14%	34%	64%	30%	59%	81%	**82%**	**82%**
	100	21%	29%	70%	30%	64%	**76%**	75%	**76%**
	150	32%	38%	72%	31%	67%	76%	77%	**78%**
	200	38%	38%	80%	44%	71%	**79%**	77%	78%
ΔR≤8	50	20%	42%	71%	42%	76%	81%	82%	**84%**
	100	25%	33%	72%	44%	74%	76%	**78%**	76%
	150	44%	46%	77%	51%	77%	**80%**	**80%**	79%
	200	42%	42%	84%	54%	77%	**82%**	79%	79%

**Table 2:** Comparison of prediction accuracy for placing task using different models, with all sensor data.

Error Distance [cm]	OTW [ms]	Model							
		SVR	DecisionTree	AdaBoost	ExtraTrees	MLP	RNN	GRU	LSTM
ΔR≤2	50	2%	23%	48%	4%	8%	**80%**	**80%**	**80%**
	100	5%	27%	41%	2%	13%	73%	**75%**	**75%**
	150	4%	16%	38%	1%	8%	74%	60%	**75%**
	200	5%	14%	37%	2%	5%	82%	**83%**	**83%**
ΔR≤4	50	6%	26%	52%	14%	28%	**80%**	**80%**	**80%**
	100	11%	28%	49%	7%	35%	75%	75%	**76%**
	150	11%	18%	43%	9%	25%	**76%**	73%	**76%**
	200	13%	17%	44%	8%	26%	83%	**84%**	83%
ΔR≤6	50	9%	30%	55%	25%	43%	80%	81%	**81%**
	100	16%	32%	54%	21%	56%	75%	75%	**77%**
	150	19%	21%	46%	26%	45%	75%	74%	**76%**
	200	21%	17%	50%	19%	51%	83%	**88%**	83%
ΔR≤8	50	16%	38%	64%	40%	58%	80%	81%	**82%**
	100	26%	38%	63%	33%	70%	75%	75%	**79%**
	150	26%	24%	52%	35%	62%	**79%**	77%	78%
	200	31%	20%	54%	33%	61%	84%	**88%**	83%

## Data Availability

Data available on request.

## Abbreviations

The following abbreviations are used in this manuscript:

ADL	Activities of daily living
AD	Anterior Deltoid
BB	Biceps Brachii
EEG	Electroencefalography
sEMG	Surface Electromyography
HMI	Human–machine interface
IMU	Inertial measurement unit
MAE	Mean absolute error
RMS	Root mean square
RMSE	Root mean square error
VAR	Variance
WL	Wavelength
OTW	Onset motion time window
MI	Mutual information
MID	Motion intention detection
MLP	Multiple Layer Perceptron
RNN	Recurrent neural network
LSTM	Long Short-Term Memory
GRU	Gated Recurrent Unit
MMG	Mechanomyographic
PM	Pectoralis Major
SVR	Support Vector Regression
TB	Triceps Brachii
